# Novel hits for acetylcholinesterase inhibition derived by docking-based screening on ZINC database

**DOI:** 10.1080/14756366.2018.1458031

**Published:** 2018-04-13

**Authors:** Irini Doytchinova, Mariyana Atanasova, Iva Valkova, Georgi Stavrakov, Irena Philipova, Zvetanka Zhivkova, Dimitrina Zheleva-Dimitrova, Spiro Konstantinov, Ivan Dimitrov

**Affiliations:** a Faculty of Pharmacy, Medical University of Sofia, Sofia, Bulgaria;; b Drug Design and Development Lab, Sofia Tech Park, Sofia, Bulgaria;; c Institute of Organic Chemistry with Centre of Phytochemistry, Bulgarian Academy of Sciences, Sofia, Bulgaria

**Keywords:** Virtual screening, molecular docking, ITC, PAMPA, Neuro 2A

## Abstract

The inhibition of the enzyme acetylcholinesterase (AChE) increases the levels of the neurotransmitter acetylcholine and symptomatically improves the affected cognitive function. In the present study, we searched for novel AChE inhibitors by docking-based virtual screening of the standard lead-like set of ZINC database containing more than 6 million small molecules using GOLD software. The top 10 best-scored hits were tested *in vitro* for AChE affinity, neurotoxicity, GIT and BBB permeability. The main pharmacokinetic parameters like volume of distribution, free fraction in plasma, total clearance, and half-life were predicted by previously derived models. Nine of the compounds bind to the enzyme with affinities from 0.517 to 0.735 µM, eight of them are non-toxic. All hits permeate GIT and BBB and bind extensively to plasma proteins. Most of them are low-clearance compounds. In total, seven of the 10 hits are promising for further lead optimisation. These are structures with ZINC IDs: 00220177, 44455618, 66142300, 71804814, 72065926, 96007907, and 97159977.

## Introduction

The enzyme acetylcholinesterase (AChE) is a serine protease (EC 3.1.1.7) catalysing the hydrolysis of the neurotransmitter acetylcholine (ACh) to choline and acetic acid. Low levels of ACh lead to cognitive impairment and dementia^1^. The inhibition of AChE increases the ACh levels and symptomatically improves the affected cognitive function^2^. AChE inhibitors (AChEIs) are the main drugs currently in use for treatment of Alzheimer’s disease (AD), the most common form of dementia^3^. Donepezil, galantamine, and rivastigmine are AChEIs. They have moderate affinity to AChE and provide delay in AD progression.

The binding site on AChE is deep and narrow gorge and consists of several domains: catalytic, anioinic, acylic, oxyanionic, and peripheral anionic^4–7^. The most important of them are the catalytic active site (CAS) where ACh hydrolysis happens and the peripheral anionic site (PAS) placed near the entrance of the gorge and associated with the formation of amyloid plaques^8^. Thus, AChE is a target with dual functionality: ACh hydrolysis and amyloid beta (Aβ) peptide aggregation. Because of its importance, AChE is a focus of many intensive and extensive drug discovery studies during the last two decades^9–11^. These studies could be grouped into three directions: lead optimisation of known AChEIs^12–16^, hybrids between them^17–24^ and search for new scaffolds^25–28^. A wide range of computational tools are involved in these studies^29^.

One of the most useful structure-based computational methods in the discovery of novel hits binding to a specific target is the molecular docking used solely or in combination with 2D- and 3D-QSAR, high-throughput screening, and/or machine learning methods^30^. Recently Chen et al. performed virtual screening on 263,146 entries from Specs database using a structure-based pharmacophore (SBP) derived on two tacrine hybrids with nanomolar affinity to AChE^31^. The top 50 compounds were docked in the AChE and the best 15 of them were tested *in vitro* by Ellman’s method^32^. Ten hits showed IC_50_ values below 10 µM. Dhanja et al. derived a ligand-based pharmacophore using the 3D structures of 16 known AChE inhibitors^33^. The pharmacophore was used to screen 50,000 small molecule natural compounds from ZINC database, followed by docking studies. The best two binders were analysed for molecular interactions with AChE, but have not been tested experimentally. Chen et al. conducted a virtual screening on Chemdiv compound collection, which contains 1,293,896 molecules^34^. Initially, the collection was screened by rapid overlay of chemical structures using the AChEI donepezil as a template. Then, the top 1% of the most similar structures was screened by SBP generated on the basis of donepezil-AChE X-ray complex. Finally, 24 compounds of the best hits were tested for AChE and BuChE activity *in vitro* by Ellman’s method. Among them, five new inhibitors were discovered.

In the present study, we conducted a docking-based virtual screening on ZINC database containing over 6 million biologically active small molecules in order to identify novel hits binding to AChE. The top 10 best-scored binders to AChE were tested *in vitro* for affinity to AChE, neurotoxicity, blood-brain barrier (BBB), and gastrointestinal (GI) permeability. Predictions of their physicochemical and ADME properties also were performed.

## Materials and methods

### Database and docking protocol

ZINC (www.zinc.docking.org) contains several databases of biologically active structures. We selected the Standard Lead-like database which consists of 6,053,287 small molecules with molecular weights between 250 and 350 g/mol, log *P* up to 3,5 and up to 7 rotatable bonds. The set was downloaded in March 2015. The molecules were docked into the X-ray structure of human recombinant acetylcholinesterase (*rh*AChE, pdb id: 4EY6, R = 2.40 Å)^35^. The docking simulations were performed by GOLD v. 5.1. [CCDC Ltd., Cambridge, UK] using a protocol previously optimised in terms of scoring function, rigid/flexible ligand and binding site, radius of the binding site, the presence/absence of a water molecule (HOH846) within the binding site, number of genetic algorithm (GA) runs^36–39^. The docking simulations in the present study were performed at the following settings: scoring function ChemPLP, flexible ligand, rigid protein, radius of the binding site 6 Å, no water molecule, 10 GA runs. The top 20 best-scored compounds were selected and docked five times with 100 GA runs each. The top 10 best-scored compounds from all runs were selected for tests.

### Compounds

*N*-Methyl-3-(2-oxo-1-pyridyl)-*N*-[(2-phenylphenyl)methyl]propanamide (**1**, ZINC72065926) and *N*-[3-(benzimidazol-1-yl)propyl]-3-indol-1-yl-propanamide (**2**, ZINC71804814) were purchased from AKos GmbH, Germany. 3-(6-Oxo-3-phenylpyridazin-1(6*H*)-yl)-*N*-phenethylpropanamide (**3**, ZINC00220177), 1-[3-(2-fluorophenyl)-1-methyl-pyrazol-4-yl]-*N*-(6-quinolylmethyl) methanamine (**4**, ZINC23159164), *N*^1^-((1-methyl-3-phenyl-1*H*-pyrazol-4-yl)methyl)piperidine-1,4-dicarboxamide (**6**, ZINC96007907), *N*-((1-methyl-3-(pyridin-3-yl)-1*H*-pyrazol-4-yl)methyl)-2-(quinolin-8-yl)ethanamine (**7**, ZINC97159977), 2–(2,4-dioxoquinazolin-1-yl)-*N*-[2-(3-fluorophenyl)ethyl]acetamide (**9**, ZINC08993868) and *N*-[2-(4-hydroxyphenyl)ethyl]-2-(1-isopropylindol-3-yl)acetamide (**10**, ZINC96116182) were obtained from MolPort SIA, Latvia. The purchased compounds arrived with analytical data for identity and purity. Compounds 2-indol-1-yl-*N*-[2-(8-quinolyloxy)ethyl]acetamide (**5,** ZINC44455618) and *N*-((1*S*,2*S*)-1-(4-aminophenyl)-1,3-dihydroxypropan-2-yl)-2-(4-methoxyphenyl)acetamide (**8**, ZINC66142300) were synthesised.

### Isothermal titration calorimetry (ITC) protocol

The ITC measurements^40^ were performed on NanoITC tool (TA Instruments, Lindon, UT) with 190 µL sample cell and 50 µL syringe. The lyophilised AChE from *Electrophorus electricus* (electric eel) (Sigma Aldrich, St. Luis, MO) was reconstructed in 50 mM TRIS-HCl pH 7.4 buffer to obtain ca. 1140 U/mL with the addition of 0.1% BSA as an enzyme stabilising factor, according to the manufacturer’s instructions. The tested compounds were prepared in 5 mM stock solutions in ethanol and diluted to 0.5 mM in 50 mM Tris-HCl pH 7.4 buffer. All samples were degassed prior the experiments. The AChE solution was placed into the sample cell and titrated by the tested compounds in 25 steps of 2 µL at 5 min intervals at 25 °C. The blank samples (buffer lacking AChE) were titrated at the same conditions. The corresponding *K*_d_ values were calculated using NanoAnalyze software (TA Instruments, Lindon, UT).

### Parallel artificial membrane permeability assay (PAMPA)

The intestinal and BBB permeabilities were measured by PAMPA Permeability Analyzer (pION Inc.) at the following settings: wavelength analysed 250–500 nm, pH 7.4, temperature 25 °C, permeation time 4 h, lipid formulation GIT-0 and stirring 60 rpm and BBB-1 and no stirring, respectively. The intestinal permeability was tested at three pH values: 5.0, 6.2, and 7.4, while the BBB permeability was tested at pH 7.4. The permeability was presented as *−*log*Pe*, where *Pe* is the permeability coefficient (10^−6 ^cm/s). Compounds with *−*log*Pe* below 5 are considered as highly permeable, with *−*log*P*_e_ between 5 and 6 – as medium permeable and with above 6 – as low permeable^41^. Carbamazepine, ketoprofen, and ranitidine were used as controls for intestinal permeability. Theophylline, progesterone, and propranolol were used as controls for BBB permeability^41^.

### Neurotoxicity test

Murine neuroblastoma NEURO-2 A cells (German collection DSMZ, Braunschweig, Germany) were cultivated under standard conditions: complete medium (90% DMEM, 10% heat-inactivated FBS, and 1 × non-essential amino acids); 37 °C and5% CO_2_ in fully humidified atmosphere. The cell line was kept in the logarithmic growth phase by splitting 1:4 once a week using trypsin/EDTA. About 30% of the cells grow like neuronal cells. For the experimental evaluation of the cytotoxicity NEURO-2A, cells were plated in 96-well flat-bottomed cell culture plates at the recommended density of 1 × 106 cells/25 cm^2^. After 24 h, the cells were treated with various concentrations of the investigational compounds and after 72-h incubation, a MTT-dye reduction assay was performed^42^. Briefly, at the end of incubation, a MTT stock solution (10 mg/ml in PBS) was added (10 µL/well). Plates were further incubated at 37 °C for 4 h. Next, the formazan crystals were dissolved by the addition of 110 µL/well 5% formic acid in 2-propanol (v/v). Absorption was measured at 580 nm wavelength on an automated ELISA reader Labexim LMR1. At least six wells per concentration were used, and data were processed using the GraphPad Prism 5.0 software 2.

### Calculation of physicochemical properties and prediction of pharmacokinetic (PK) parameters

The main physicochemical properties p*K*_a_, log*P*, log*D*_7.4_, polar surface area (*PSA*), number of hydrogen-bond donors (*HBD*), and hydrogen-bond acceptors (*HBA*) in the molecules of the tested compounds were calculated using ACD/log*D* v. 9.08 (ACD Inc., Canada). The fraction ionised as a base at pH = 7.4 (*f*_B_) was calculated according to the equation:

$$f_{B}=\frac{1}{1+10^{\left(7.4-{pK}_{a}\right)}}.$$

The key PK parameters were predicted by quantitative structure–activity relationships (QSPkRs) models, derived previously^43–46^. Briefly, the PK parameters of 145 neutral and/or 262 basic drugs^47^ were used to derive QSPkR models by multiple linear regression (MLR) with MDL QSAR v. 2.2 (MDL Information Systems Inc., San Leandro, CA). Three PK parameters were modelled: the steady-state volume of distribution (VD^ss^), free fraction of drug in plasma (*f*_u_), and unbound clearance (CL_u_). The total clearance (CL) and half-life (*t*_1/2_) were calculated following the equations.

$$\text{CL}={\text{CL}}_{\text{u}}\times f_{\text{u}}$$

$$t_{1/2}=\frac{\ln2\times {\text{VD}}^{\text{ss}}}{\text{CL}}$$

The models are statistically significant and meet the criteria for good performing QSPkRs^48^^,^^49^. The AChEI galantamine (GAL) is given as a reference compound.

## Results

### Docking-based screening of ZINC database on rhAChE

**Figure 1.**
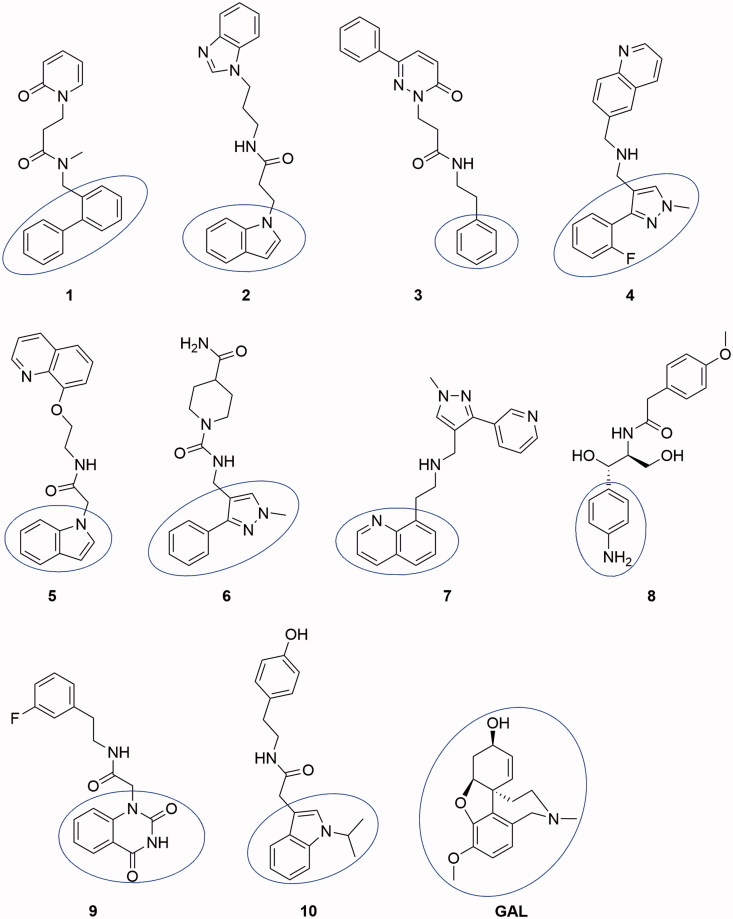
Structures of the top 10 best-scored hits. The fragments binding in CAS are given by ellipse.

The top 10 best-scored hits by ChemPLP from the docking of 6,053,287 molecules from ZINC database on *rh*AChE are given in Figure 1. The dockings were performed with flexible ligands and rigid binding site lacking the water molecule necessary for the binding of GAL. The ChemPLP scores are given in Table 1.

**Table 1.** Docking score, *ee*AChE affinity, neurotoxicity, BBB, and GIT permeability of the tested compounds.

ID	ZINC ID	ChemPLP score	*K*_d_ μM ITC	IC_50_ μM Neuro2A	*pP*_e_ pH 7.4 BBB PAMPA	*pP*_e_ pH 6.2 GIT PAMPA
1	72065926	104.58	0.525	>100	4.540	4.268
2	71804814	101.17	0.618	>100	4.452	4.339
3	00220177	101.11	0.555	>100	4.294	4.207
4	23159164	100.79	0.618	35	4.258	4.251
5	44455618	99.89	0.517	>100	4.146	4.230
6	96007907	99.64	0.613	>100	5.008	4.428
7	97159977	99.62	0.577	>100	4.267	4.246
8	66142300	99.02	0.682	>100	4.731	4.469
9	08993868	98.69	Non-binder	>100	4.628	4.361
10	96116182	98.64	0.735	53	4.311	4.202
GAL		74.560^39^	388.2	>50^39^	5.060^39^	4.268

Compounds with *pPe* < 5 are considered as highly permeable, with *pPe* between 5 and 6 – as medium permeable and with *pPe* > 6 – as low permeable.

All compounds consisted of two aromatic moieties connected by a linker of 3–7 carbon chain containing NH or NHCO group. An exception is made by compound **6** which contain one non-aromatic piperidine ring in the linker. The docking poses showed that the first aromatic moiety binds in CAS, the aliphatic chain is stretching along the binding gorge and the second aromatic ring binds in PAS (Figure 1).

Compounds **1**–**4**, **6**, **7**, **9**, and **10** were purchased; compounds **5** and **8** were synthesised.

### Synthesis of compounds 5 and 8

**Scheme 1.**
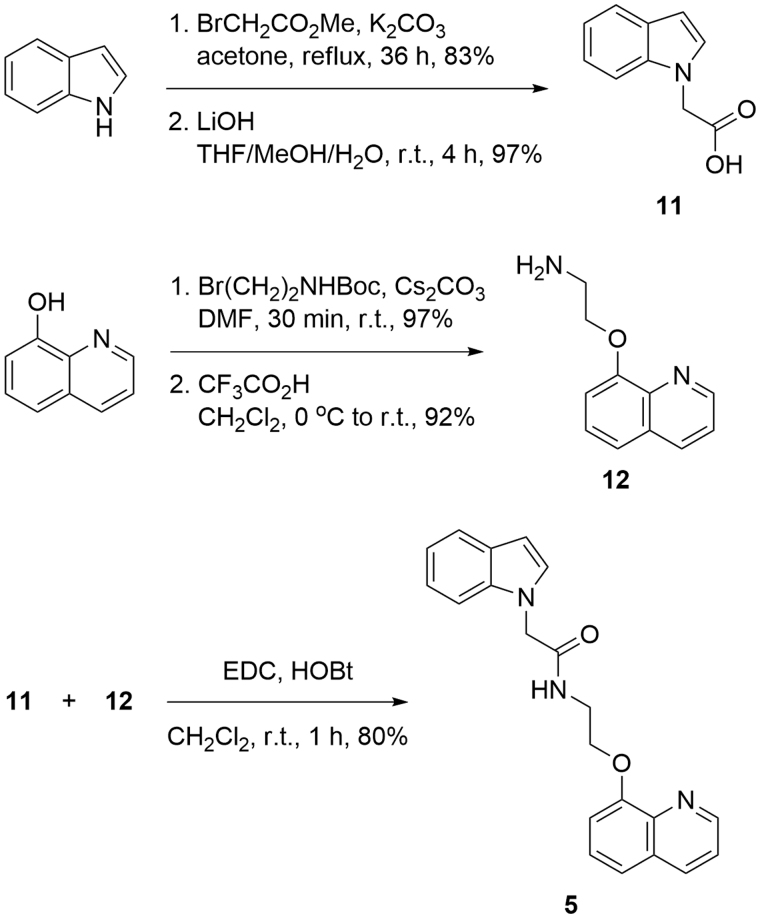
Synthesis of compound **5**.

The synthetic strategy towards the synthesis of compound **5** was based on the formation of amide bond between 2-indoleacetic acid **11** and quinoline derived amine **12** (Scheme 1).

The desired acid **11** was synthesised via nucleophilic substitution of methyl 2-bromoacetate with deprotonated indole, followed by hydrolysis of the resulting methyl ester^50^. Reaction of 8-hydroxyquinoline with *N*-Boc protected 2-bromoethanamine in the presence of Cs_2_CO_3_ as a base, and subsequent deprotection of the amine afforded 2-(quinolin-8-yloxy)ethanamine **12** in excellent yield. The coupling between the two building blocks was achieved applying 1-ethyl-3-(3-dimethylaminopropyl)carbodiimide (EDC) and 1-Hydroxybenzotriazole hydrate (HOBT) in dichloromethane (Scheme 1).

**Scheme 2.**
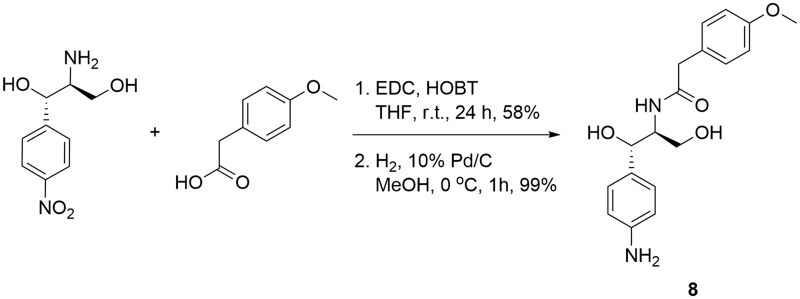
Synthesis of compound **8**.

Compound **8** was synthesised by coupling of the commercially available (1*S*,2*S*)-2-amino-1-(4-nitrophenyl)propane-1,3-diol with 4-methoxyphenylacetic acid and subsequent catalytic hydrogenation of the nitro group to the corresponding amine (Scheme 2).

The detailed synthesis, analytical data (incl. LC-MS and elemental analyses) and copies of ^1^H and ^13^C NMR spectra for compounds **5** and **8** are given in the Supplementary File.

### Estimation of binding affinity by ITC

The binding affinity of the best-scored compounds was tested *in vitro* by ITC as described in “Materials and methods” section. AChE from electric eel (*ee*AChE) was used in the measurements. The UniProt alignment of *rh*AChE (UniProt: P22303) and *еe*AChЕ (UniProt: O42275) have showed that all 17 residues forming the binding gorges are identical^39^. Our previous experience has shown that the docking scores predicted for *rh*AChE correlate well with the experimental binding affinity measured on *ee*AChE^36–39^. The *K*_d_ values of the tested compounds are given in Table 1. Compound **9** does not interact with *ee*AChE. For the rest, the *K*_d_s are in the range of 0.517–0.735 µM.

### Estimation of GIT and BBB permeability by PAMPA

The permeability of the compounds tested by PAMPA shows that all of them are highly permeable through the GIT and BBB (Table 1). Only compound **6** has intermediate BBB permeability with *−*log*Pe* = 5.008. The *−*log*Pe* for the rest compounds ranges from 4.146 to 4.731 for the BBB permeability and from 4.202 to 4.469 for the GIT permeability at pH = 6.2. For comparison, GAL has *−*log*Pe* 5.060 and 4.268 for BBB and GIT permeability, respectively^39^, which corresponds well to its low ability to penetrate BBB by passive diffusion^51^ and to 90% oral bioavailability^52^. Moderate correlations exist between log*D*_7.4_ and BBB permeability (*r* = 0.693) and between log*D*_7.4_ and GIT permeability (*r* = 0.637) of the tested compounds.

### Estimation of neurotoxicity on neuro-2A cells

The neurotoxicity of the compounds was tested on NEURO-2A cells as described in “Materials and methods” section. All of them are non-toxic at concentrations up to 100 µM, apart from compounds **4** and **10**. Compound **4** has IC_50_ = 35 µM and compound **10** has IC_50_ = 53 µM.

### Physicochemical properties and PK parameters

The molecular weights of the tested compounds vary in a short range from 330.38 to 347.41 g/mol. The p*K*_a_ values are from −0.69 to 8.14, log*P*s are between 0.13 and 3.65, log*D*_7.4 _s – between 0.13 and 3.42 (Table 2). According to *f*_B_, compounds **4** and **7** are moderate bases, partially ionised at physiological pH, and the rest are neutral molecules. Most of the compounds are lipophilic, only compounds **6** and **8** are rather hydrophilic with *PSA*s above 80 Å^2^. The number of *HBD*s in the molecules is up to 5 and the number of *HBA*s – up to 10.

**Table 2.** Calculated physicochemical properties and predicted PK parameters of the tested compounds. VD^ss^ is the volume of distribution in steady state, *f*_u_ – the fraction of free (unbound) compound in plasma, CL – total plasma clearance, *t*_1/2_ – half-life.

ID	MW (g/mol)	p*K*_a_	log*P*	log*D*_7.4_	PSA Å^2^	HBD	HBA	*f*_B_	VD^ss^ (L/kg)	*f*_u_	CL (mL/min/kg)	*t*_1/2_ (h)
1	346.42	0.14	2.69	2.69	40.62	0	4	0.00	1.09	0.04	0.59	21.23
2	346.43	5.71	3.18	3.17	51.85	1	5	0.02	1.34	0.02	142.66	0.11
3	347.41	−0.69	1.96	1.96	61.77	1	5	0.00	1.29	0.16	2.48	6.02
4	346.40	7.29	3.65	3.42	42.74	1	4	0.44	3.17	0.03	5.47	6.69
5	345.39	3.25	2.82	2.82	56.15	1	5	0.00	0.96	0.01	9.82	1.13
6	341.41	2.09	0.15	0.15	93.25	3	7	0.00	0.84	0.27	1.28	7.57
7	343.43	8.14	1.98	1.21	55.63	1	5	0.85	3.67	0.06	5.45	7.78
8	330.38	4.46	0.13	0.13	104.81	5	6	0.00	0.73	0.14	0.59	14.27
9	341.34	−0.83	1.79	1.79	78.51	2	6	0.00	1.35	0.09	1.68	9.27
10	336.43	−0.67	3.18	3.18	54.26	2	4	0.00	1.61	0.02	8.30	2.24
GAL	287.35	7.92	1.75	1.12	41.93	1	4	0.77	2.30^47^	0.83^47^	5.60^47^	5.30^47^

The predicted VD^ss^ values of the bases **4** and **7** are 3.17 L/kg and 3.67 L/kg, respectively. They are considerably higher than the VD^ss^s of the neutral molecules, which range from 0.73 to 1.61 L/kg. This is in a good agreement with the distribution of VD^ss^ of the drugs from the training set of bases used to derive the model^41^. The VD^ss^s in the training set range between 0.073 and 140 L/kg, with mean 5.90 L/kg and median 2.45 L/kg. The lower VD^ss^s of the neutral molecules also is in accordance with the VD^ss^s of the training set of neutral molecules, which vary between 0.16 and 25 L/kg, with mean 1.94 L/kg and median 1 L/kg^44^. As log*P* is one of the descriptors with high positive effect on VD^ss43^, the hydrophilic compounds **6** and **8** have reasonably the lowest VD^ss^s.

The tested compounds bind extensively to plasma proteins (PP). The predicted *f*_u_s are between 0.01 and 0.27, i.e. 73–99% of the compounds are bound to plasma proteins. Again, lipophilicity is the major governing factor for high PP binding^44^. The hydrophilic compounds **6** and **8** have the highest *f*_u_s.

The predicted CL values vary between 0.59 and 9.82 mL/min/kg. The CL of compound **2** is overpredicted, as it exceeds the maximum total body blood flow (80 mL/min/kg). Most of the tested compounds are low-clearance compounds with CLs below 30% of hepatic blood flow (*Q*_H_ = 21 mL/min/kg)^53^^,^^54^. Only compounds **5** and **10** are moderate-clearance drugs with CLs around 50% of *Q*_H_.

The half-lives *t*_1/2_ were predicted from the corresponding VD^ss^ and CL values. Expectedly, the *t*_1/2_ of compound **2** is underpredicted. Compounds **5** and **10** have very short *t*_1/2_ due to the low VD^ss^ and relatively high CL. The rest of compounds have moderate *t*_1/2_ in the range of 6–21 h, allowing convenient multiple-dose regimens.

## Discussion

The standard lead-like set of ZINC database was virtually screened by molecular docking on *rh*AChE and the 10 best-scored structures were tested *in vitro* for binding affinity to the enzyme, neurotoxicity, permeability across GIT and BBB. ADME properties were predicted. Nine of the compounds bind well the enzyme with *K*_d_ in nanomolar range, eight of them are non-toxic at concentrations up to 100 µM. All of the tested compounds are highly permeable across the GIT and BBB, have *MW*s up to 350 g/mol, log*D*_7.4_ up to 3.5 and bind extensively to plasma proteins. Most of them are low-clearance compounds.

**Figure 2.**
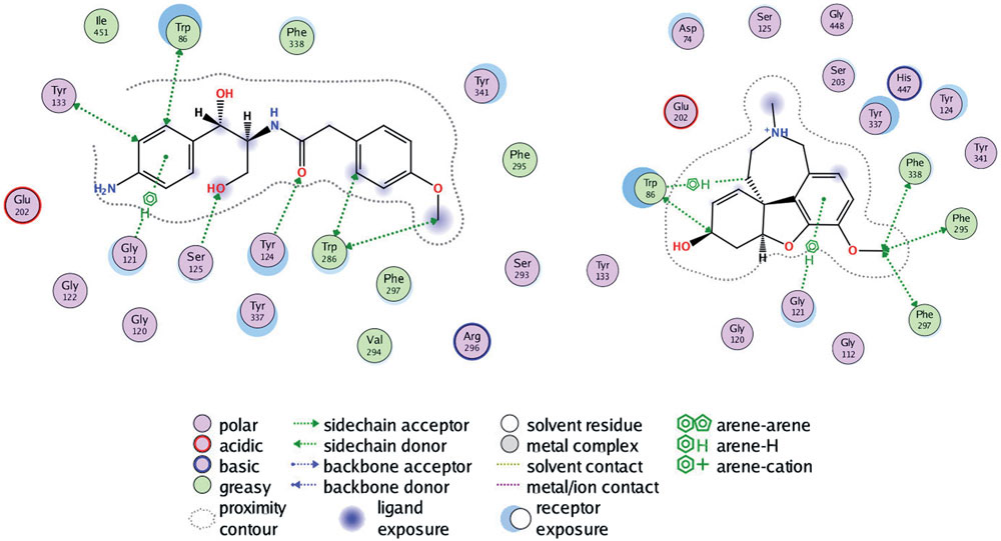
Continued.

Compound **1** is a neutral molecule with log*D*_7.4_ of 2.69 and high affinity to AChE (*K*_d_ = 525 nM). It is non-toxic on Neuro-2 A cells, permeates easily the gastric mucosa and the BBB, 96% of the molecules are bound to plasma proteins. Although the VD^ss^ is 1.09 L/kg, compound **1** is cleared very slowly (CL = 0.59 mL/min/kg) and has long half-life (*t*_1/2_ = 21.23 h). The docked pose of **1** into *rh*AChE shows that the biphenyl fragment binds in CAS, while the oxo-pyridyl moiety is placed in PAS (Figure 2). Hydrogen bonds are formed between Tyr337 and carbonyl oxygen atom from the linker and between His447 and the carbonyl oxygen atom from the oxo-pyridyl moiety. Hydrogen–π interactions exist between Trp86, Gly121, and biphenyl, and between Phe295 and oxo-pyridyl. Trp286, Val294, Phe297, Phe338, and His447 take part in a network of hydrophobic interactions with the linker and the oxo-pyridyl fragment.

Figure 2.Interactions between rhAChE residues and (1) compound **1**, (2) compound **2**, (3) compound **3**, (4) compound **5**, (5) compound **6**, (6) compound **7**, (7) compound **8**, and (8) GAL in the docked complex.

**Figure:**
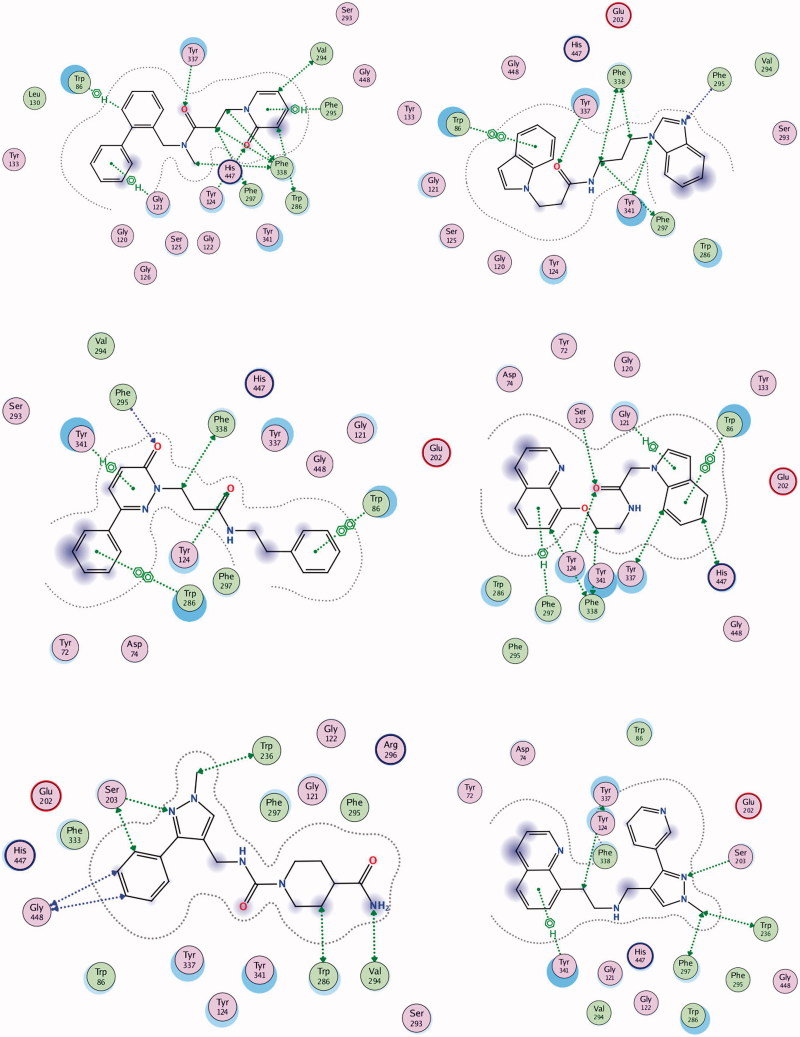

Compound **2** is a weak base with log*D*_7.4_ of 3.17, high affinity to AChE (*K*_d_ = 618 nM), good permeability and absence of toxicity. The predicted ADME properties show that 98% of compound **2** are bound to plasma proteins and the VD^ss^ is 1.34 L/kg. Compound **2** has very intensive probably overpredicted clearance (CL =143 mL/min/kg) and ultra-short half-life (*t*_1/2_ = 0.11 h). The indole moiety stacks with Trp86 in CAS, while the benzimidazol fragment is positioned in PAS making a hydrogen bond with Phe295 (Figure 2). Another hydrogen bond is formed between Tyr337 and the carbonyl oxygen atom from the linker. Phe297, Tyr341, and Phe338 are involved in hydrophobic interactions with the linker.

Compound **3** is a high binder to AChE with *K*_d_ = 555 nM, log*D*_7.4_ of 1.96, VD^ss^ of 1.29 L/kg, moderate clearance (CL =15.48 mL/min/kg) and half-life of 6 h. It is permeable and non-toxic. The single phenyl ring of **3** binds in CAS stacking with Trp86, while the phenylpiperazine moiety is placed in PAS interacting with Trp286 and Tyr341 and forming a hydrogen bond with Phe295 (Figure 2). Compound **4** binds well to the enzyme with *K*_d_ = 618 nM but because of its toxicity (IC_50_ = 35 µM), it is not considered as a perspective hit for further optimisation.

Compound **5** is a neutral molecule with log*D*_7.4_ of 2.82 and *K*_d_ of 517 nM. It is non-toxic, easily permeable with VD^ss^ = 0.96 L/kg, binds extensively (99%) with plasma proteins, is cleared with moderate rate (CL =9.82 mL/min/kg) and has short half-life (*t*_1/2_ = 1.13 h). The indolyl fragment inhibits CAS making π–π interaction with Trp86 and hydrogen–π bond with Gly121, while the quinolyl moiety binds in PAS forming hydrogen–π bond with Phe297 (Figure 2). The carbonyl oxygen from the linker makes hydrogen bonds with Tyr124 and Ser125.

Compound **6** binds tightly to *ee*AChE with *K*_d_ of 613 nM. It is a neutral molecule with log*D*_7.4_ of 0.15, VD^ss^ = 0.84 L/kg, 73% is bound to plasma proteins, has low clearance (CL =1.28 mL/min/kg) and half-life of 7.57 h. The phenyl-pyrazolyl moiety is bound in CAS making hydrogen bond with Ser203, while the piperidine fragment is oriented towards the PAS and binds hydrophobically to Trp286 and Val294 (Figure 2).

Compound **7** is a weak base partially protonated at pH 7.4 with log*D*_7.4_ of 1.21. Binds to AChE with *K*_d_ of 577 nM. It absorbs easily through the intestinal mucosa, crosses the BBB, distributes extensively with VD^ss^ of 3.67 L/kg, is cleared moderately (CL = 5.45 mL/min/kg) and has half-life of 7.78 h. The quinolyl fragment enters CAS interacting with Tyr341, while the pyridyl-pyrazolyl moiety makes hydrogen bond with Ser203 near PAS (Figure 2).

Compound **8** binds to *ee*AChE with *K*_d_ = 682 nM. It is a neutral molecule with small VD^ss^, slow clearance (CL = 0.59 mL/min/kg) and half-life of 14.27 h. The aminophenyl binds in CAS, while the methoxy-benzene is placed in PAS. Hydrogen bonds are formed between Ser125 and oxygen from the CH_2_OH group and between Tyr124 and the carbonyl oxygen from the linker (Figure 2). Compound **9** is a non-binder and compound **10** is toxic on Neuro-2A (IC_50_ = 53 nM). Both of them are not considered as prospective hits for AChE inhibition.

GAL is a well-known AChE inhibitor. The *K*_d_ found by ITC is 388.2 µM. It is a weak base with p*K*_a_ = 7.92, partially protonated at pH 7.4. Most of it (83%) exists as a free, non-bound fraction in plasma, distributed extensively with VD^ss^ of 2.30 L/kg, has moderate clearance (CL = 5.60 mL/min/kg) and half-life of 5.30 h^47^. GAL easily crosses the intestinal mucosa (*pPe* GIT =5.060) which corresponds well to its oral bioavailability of 90%^52^. The value of 5.060 for *pPe* BBB, found in our previous study^39^, indicates for moderate ability to cross the BBB by passive diffusion but mediation by choline transport system has been suggested^55^. GAL binds mainly in CAS; only the methoxy group interacts with Phe295, Phe297 and Phe338 from PAS. The pose presented in Figure 2 has RMSD of 1.0396 Å from the X-ray data of the complex GAL-AChE^35^ which is a good validation of the docking protocol used in the present study.

## Conclusions

The virtual screening on ZINC database and the following *in vitro* tests and PK predictions featured seven new hits for acetylcholinesterase inhibition (compounds **1**, **2**, **3**, **5**, **6**, **7**, and **8**). The hits are non-toxic, GIT and BBB permeable and bind the enzyme AChE with nanomolar affinity. They could be considered for further lead optimisation.

## Supplementary Material

IENZ_1458031_Supplementary_Materials.pdf
