# Supplementary material for: Novel hits for acetylcholinesterase inhibition derived by docking-based screening on ZINC database
Source: J Enzyme Inhib Med Chem. 2018 Apr 13;33(1):768–76. doi: 10.1080/14756366.2018.1458031 (PMC6010092; doi:10.1080/14756366.2018.1458031)
Supplement: IENZ_1458031_Supplementary_Materials.pdf [file IENZ_A_1458031_SM2387.pdf]

Supplemental File

Novel hits for acetylcholinesterase inhibition derived by docking-based screening on ZINC database

Contents:

1. Synthesis and analytical data for compounds **5** and **8**.
2. Copies of ^{1}H and ^{13}C NMR spectra for compounds **5** and **8**.

1. Synthesis and analytical data for compounds **5** and **8**.

- **General**

Reagents were commercial grade and used without further purification. Indole, methyl bromoacetate, 8-hydroxyquinoline, tert-butyl *N*-(2-bromoethyl)carbamate, (1*S*,2*S*)-2-Amino-1-(4-nitrophenyl)propane-1,3-diol and 4-Methoxyphenylacetic acid were purchased from Acros Organics. Thin layer chromatography (TLC) was performed on aluminum sheets pre-coated with Merck Kieselgel 60 F254 0.25 mm (Merck). Flash column chromatography was carried out using Silica Gel 60 230-400 mesh (Fluka). Commercially available solvents were used for reactions, TLC and column chromatography. Melting points were determined in a capillary tube on SRS MPA100 OptiMelt (Sunnyvale, CA, USA) automated melting point system (uncorrected). The NMR spectra were recorded on a Bruker Avance II+ 600 (600.13 for ^{1}H MHz and 150.92 MHz for ^{13}C NMR) spectrometer with TMS as internal standards for chemical shifts (δ, ppm). ^{1}H and ^{13}C NMR data are reported as follows: chemical shift, multiplicity (s = singlet, d = doublet, t = triplet, q = quartet, br = broad, m = multiplet), coupling constants (Hz), integration, identification. The assignment of the ^{1}H and ^{13}C NMR spectra was made on the basis of COSY and HSQC experiments. LC–MS analyses were performed using a Q Exactive Plus Orbitrap Mass spectrometer (Thermo Fisher Scientific, Bremen, Germany), equipped with an electrospray (ESI) probe. The spectra were recorded on a positive mode using a MS Full Scan mode. Elemental analyses were performed using Vario EL3 CHNS(O).

- Synthesis of 2-(1*H*-indol-1-yl)acetic acid **11**.

To a solution of indole (0.400 g, 3.41 mmol) in acetone (10 ml) was added potassium carbonate (0.943 g, 6.82 mmol) and methyl bromoacetate (0.48 mL, 5.12 mmol). The mixture was heated to reflux for 36 hours and then the solvent was concentrated under reduced pressure. The reidue was purified *via* flash column chromatography on silica gel (petroleum ether/EtOAc = 10:1) to give methyl 2-(1*H*-indol-1-yl)acetate as yellowish oil. Yield: 83%. ^{1}H NMR ($CDCl_3$, 600 MHz) δ = 7.64 (d, *J* = 7.9 Hz, 1H, Arom), 7.26-7.21 (m, 2H, Arom), 7.15-7.12 (m, 1H, Arom), 7.09 (d, *J* = 3.2 Hz, 1H, Arom), 6.57 (d, *J* = 3.2 Hz, 1H, Arom), 4.86 (s, 2H, N-CH_2), 3.74 (s, 3H, OCH_3) ppm. ^{13}C NMR ($CDCl_3$, 150.9 MHz) δ = 169.06 (CO), 136.40 (C-Arom), 128.55 (C-Arom),

128.39 (CH-Arom), 122.07 (CH-Arom), 121.13 (CH-Arom), 119.89 (CH-Arom), 108.84 (CH-Arom), 102.53 (CH-Arom), 52.53 (OCH_3), 47.68 (N-CH_2) ppm. HRMS found for $C_{11}H_{12}NO_2^+$: m/z 190.0859 $[M+H]^+$, calcd. m/z 190.0863.

To a solution of the methyl ester (0.538 g, 2.84 mmol) in 21 ml THF/MeOH/H_2O (1/1/1) was added LiOH.H_2O (0.358 g, 8.53 mmol). The mixture was stirred for 1 hour at room temperature and then was acidified with 2N HCl, extracted with EtOAc and concentrated to afford 2-(1H-indol-1-yl)acetic acid **11**, which was used without further purification.

- Synthesis of 2-(quinolin-8-yloxy)ethanamine **12**.

To a solution of 8-hydroxyquinoline (0.086 g, 0.60 mmol) in DMF (2 ml) was added cesium carbonate (0.388 g, 1.20 mmol). The mixture was stirred for 5 min at room temperature and a solution of tert-butyl *N*-(2-bromoethyl)carbamate (0.200 g, 0.89 mmol) in DMF (3 ml) was added. After 30 min at room temperature the reaction was quenched with water and extracted with EtOAc. The organic layers were washed with water, dried and concentrated. The product was purified *via* flash column chromatography on silica gel (petroleum ether/EtOAc = 1:2) and crystallized from petroleum ether/Et_2O to give 0.166 g tert-butyl 2-(quinolin-8-yloxy)ethylcarbamate as white crystals, m.p. 112-115 °C. Yield: 97%. ^{1}H NMR ($CDCl_3$, 600 MHz) δ = 9.03 (d, *J* = 3.1 Hz, 1H, Arom), 8.19 (dd, *J* = 8.2; 0.9 Hz, 1H, Arom), 7.49-7.46 (m, 2H, Arom), 7.43 (d, *J* = 8.1 Hz, 1H, Arom), 7.08 (d, *J* = 7.6 Hz, 1H, Arom), 6.23 (br, 1H, NH), 4.25 (t, *J* = 4.8 Hz, 2H, OCH_2), 3.72 (t, *J* = 5.1 Hz, 2H, N-CH_2), 1.45 (s, 9H, $OC(CH_3)_3$) ppm. ^{13}C NMR ($CDCl_3$, 150.9 MHz) δ = 173.65 (CO), 154.13 (C-Arom), 148.92 (CH-Arom), 139.43 (C-Arom), 136.68 (C-Arom), 129.51 (CH-Arom), 126.95 (CH-Arom), 121.74 (CH-Arom), 120.00 (CH-Arom), 109.41 (CH-Arom), 79.17 ($O\mathit{C}(CH_3)_3$), 68.73 ($O\mathit{C}H_2$), 39.90 ($N\mathit{C}H_2$), 28.39 ($OC(\mathit{C}H_3)_3$) ppm. HRMS found for $C_{16}H_{21}N_2O_3^+$: m/z 289.1538 $[M+H]^+$, calcd. m/z 289.1547.

To a solution of tert-butyl 2-(quinolin-8-yloxy)ethylcarbamate (0.037 g, 0.13 mmol) in dichloromethane (4 ml) was added CF_3CO_2H (0.65 ml) dropwise at 0 °C. The mixture was stirred for 45 min at r.t. and quenched by dropwise addition to aq $NaHCO_3$. The mixture was additionally alkalized with aq. KOH and extracted with CH_2Cl_2/MeOH (10:1). The organic layers dried and concentrated to give crude 2-(quinolin-8-yloxy)ethanamine **12** (0.022 g), which was used without further purification. Yield: 92%. ^{1}H NMR ($CDCl_3$, 600 MHz) δ = 8.94 (d, *J* = 2.7 Hz, 1H, Arom), 8.15 (dd, *J* = 8.3; 1.2 Hz, 1H, Arom), 7.47-7.44 (m, 2H, Arom), 7.42 (d, *J* = 8.0 Hz, 1H, Arom), 7.08 (d, *J* = 7.5 Hz, 1H, Arom), 4.29 (s, 2H, OCH_2), 3.35 (s, 2H, N-CH_2) ppm. ^{13}C NMR ($CDCl_3$, 150.9 MHz) δ = 154.46 (C-Arom), 149.19 (CH-Arom), 140.10 (C-Arom), 136.18 (C-Arom), 129.51 (CH-Arom), 126.75 (CH-Arom), 121.69 (CH-Arom), 119.94 (CH-Arom), 109.06 (CH-Arom), 70.23 ($O\mathit{C}H_2$), 41.04 ($N\mathit{C}H_2$) ppm. HRMS found for $C_{11}H_{13}N_2O^+$: m/z 189.1019 $[M+H]^+$, calcd. m/z 189.1022.

- Synthesis of 2-indol-1-yl-*N*-[2-(8-quinolyloxy)ethyl]acetamide **5**.

To a solution of acid **11** (0.023 g, 0.13 mmol) in CH_2Cl_2 (3 ml) was added EDC (0.025 g, 0.13 mmol), HOBT (0.018 g, 0.13 mmol) and amine **12** (0.022 g, 0.12 mmol). The mixture was stirred for 1 hour at r.t. and the product formation was monitored by TLC. The mixture was concentrated till dry and directly subjected to flash column chromatography on silica gel (CH_2Cl_2/MeOH = 50:1) to give the desired amide (0.032 g) as white amorphous solid, m.p. 139-141 °C. Yield: 80%. ^{1}H NMR ($CDCl_3$, 600 MHz) δ = 8.56 (dd, *J* = 4.2; 1.7 Hz, 1H, quin.), 8.13

(dd, J = 8.3; 1.6 Hz, 1H, quin.), 7.51 (d, J = 7.9 Hz, 1H, ind.), 7.45-7.38 (m, 4H, NH, quin.), 7.12 (d, J = 8.2 Hz, 1H, ind.), 7.04 (d, J = 3.2 Hz, 1H, ind.), 6.99 (d, J = 7.1 Hz, 1H, quin.), 6.89 (t, J = 7.1 Hz, 1H, ind.), 6.83 (t, J = 7.8 Hz, 1H, ind.), 6.50 (d, J = 3.1 Hz, 1H, ind.), 4.79 (s, 2H, NCH_2CO), 4.21 (t, J = 4.9 Hz, OCH_2), 3.76 (t, J = 5.3 Hz, NCH_2) ppm. ^{13}C NMR ($CDCl_3$, 150.9 MHz) δ = 168.56 (CO), 154.07 (C), 148.72 (CH), 139.87 (C), 136.27 (C), 136.15 (CH), 129.38 (C), 128.49 (CH, C), 126.74 (CH), 122.15 (CH), 121.53 (CH), 120.86 (CH), 120.23 (CH), 119.98 (CH), 109.91 (CH), 109.04 (CH), 102.75 (CH), 68.03 (OCH_2), 50.16 (N*C*H_2CO), 38.78 (CONHCH_2) ppm. HRMS found for $C_{21}H_{20}N_3O_2^+$: m/z 346.1540 [M+H]$^+$, calcd. m/z 346.1550. Elemental analyses for $C_{21}H_{19}N_3O_2$ (345.39): calcd. C 73.03; H 5.54; N 12.17, found C 73.16, H 5.11, N 12.25.

- Synthesis of *N*-((1*S*,2*S*)-1-(4-aminophenyl)-1,3-dihydroxypropan-2-yl)-2-(4-methoxyphenyl)acetamide **8**.

To a solution of 4-Methoxyphenylacetic acid (0.078 g, 0.47 mmol) in THF (10 ml) was added EDC (0.099 g, 0.52 mmol), HOBT (0.070 g, 0.52 mmol) and (1*S*,2*S*)-2-amino-1-(4-nitrophenyl)propane-1,3-diol (0.100 g, 0.47 mmol). The mixture was stirred for 2 hours at r.t. and the product formation was monitored by TLC. The mixture was concentrated till dry and diluted with CH_2Cl_2. The solution was washed with 1N HCl followed by water, dried and concentrated. The product crystallized from CH_2Cl_2, m.p. 136-139 °C. The crystals were filtered and washed with CH_2Cl_2 to give *N*-((1*S*,2*S*)-1,3-dihydroxy-1-(4-nitrophenyl)propan-2-yl)-2-(4-methoxyphenyl)acetamide in 58% yield. ^{1}H NMR ($CDCl_3/CD_3OD$ = 5:1, 600 MHz) δ = 8.06 (d, J = 8.3 Hz, 2H, Arom.), 7.39 (d, J = 8.3 Hz, 2H, Arom.), 6.98 (d, J = 8.2 Hz, 2H, Arom.), 6.82 (d, J = 8.2 Hz, 2H, Arom.), 5.11 (s, 1H, C*H*OH), 4.08-4.05 (m, 1H, C*H*NH), 3.83 (s, 3H, OCH_3), 3.70-3.63 (m, 2H, C*H*$_2$OH), 3.37-3.30 (m, 2H, $PhCH_2$) ppm. ^{13}C NMR ($CDCl_3/CD_3OD$ = 5:1, 150.9 MHz) δ = 172.52 (CO), 158.49 (C), 149.44 (C), 146.65 (C), 129.83 (2CH), 126.35 (2CH, C), 122.92 (2CH), 113.82 (2CH), 69.92 (*C*HOH), 61.23 (*C*H_2OH), 55.79 (*C*HNHCO), 54.95 (OCH_3), 42.08 (*C*H_2Ph) ppm. HRMS found for $C_{18}H_{21}N_2O_6^+$: m/z 361.1385 [M+H]$^+$, calcd. m/z 361.1394.

The *N*-((1*S*,2*S*)-1,3-dihydroxy-1-(4-nitrophenyl)propan-2-yl)-2-(4-methoxyphenyl)acetamide (0.083 g, 0.23 mmol) was dissolved in MeOH (10 ml), flashed with Ar and cooled to 0 °C. 10% Pd/C was added at one portion and the atmosphere was changed to H_2 with the help of a hydrogen filled balloon. The mixture was monitored by TLC and after 1 hour at room temperature no traces of the starting compound were detected. The mixture was filtered through a pad of Celite and concentrated till dry. Crystallization from petroleum ether/Et_2O gave the desired product **8** as white crystals, m.p. 150-153 °C. Yield: 99%. ^{1}H NMR (CD_3OD, 600 MHz) δ = 7.06 (d, J = 8.6 Hz, 2H, Arom.), 6.97 (d, J = 8.3 Hz, 2H, Arom.), 6.82 (d, J = 8.7 Hz, 2H, Arom.), 6.62 (d, J = 8.4 Hz, 2H, Arom.), 4.74 (d, J = 4.3 Hz, 1H, C*H*OH), 3.98-3.95 (m, 1H, C*H*NHCO), 3.74 (s, 3H, OCH_3), 3.60 (dd, J = 10.9; 6.2 Hz, 1H, C*H*$_2$OH), 3.40 (dd, J = 10.9; 5.8 Hz, 1H, C*H*$_2$OH), 3.42 (d, J = 14.8 Hz, 1H, $PhCH_2$), 3.36 (d, J = 14.8 Hz, 1H, $PhCH_2$) ppm. ^{13}C NMR (CD_3OD, 150.9 MHz) δ = 173.37 (CO), 158.69 (C), 146.45 (C), 131.57 (C), 130.01 (2CH), 127.23 (C), 126.80 (2CH), 115.09 (2CH), 113.65 (2CH), 71.13 (*C*HOH), 61.08 (*C*H_2OH), 56.94 (*C*HNHCO), 54.95 (OCH_3), 41.65 (*C*H_2Ph) ppm. HRMS found for $C_{18}H_{23}N_2O_4^+$: m/z 331.1646 [M+H]$^+$, calcd. m/z 331.1652. Elemental analyses for $C_{18}H_{22}N_2O_4$ (330.38): calcd. C 65.44; H 6.71; N 8.48, found C 65.13, H 6.35, N 8.41.

2. Copies of ^{1}H and ^{13}C NMR spectra for compounds **5** and **8**.

Compound 5:

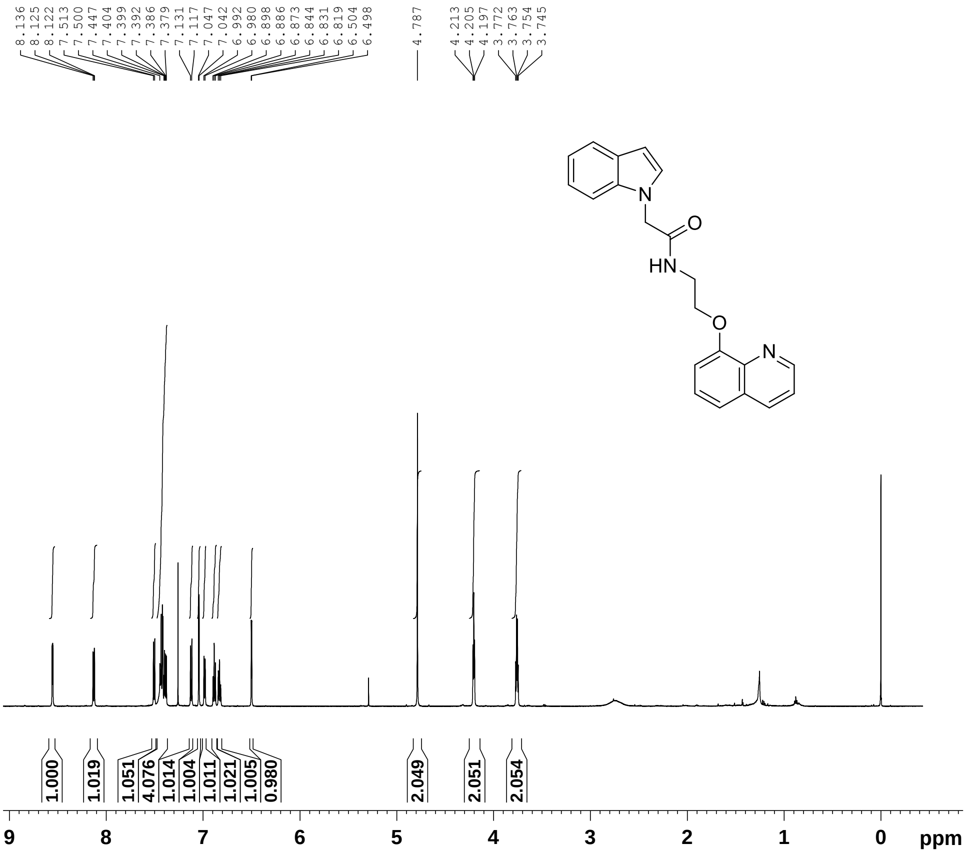

NAME FJ03902
EXPNO 11
PROCNO 1
Date_ 20170518
Time 20.47 h
INSTRUM spect
PROBHD Z847801_0047 (
PULPROG zg30
TD 32768
SOLVENT CDCl3
NS 1
DS 0
SWH 9615.385 Hz
FIDRES 0.586877 Hz
AQ 1.7039860 sec
RG 161
DW 52.000 usec
DE 13.95 usec
TE 293.0 K
D1 1.00000000 sec
TD0 1
SFO1 600.0145608 MHz
NUC1 1H
P1 10.85 usec
SI 65536
SF 600.0100145 MHz
WDW EM
SSB 0
LB 0.00 Hz
GB 0
PC 1.00

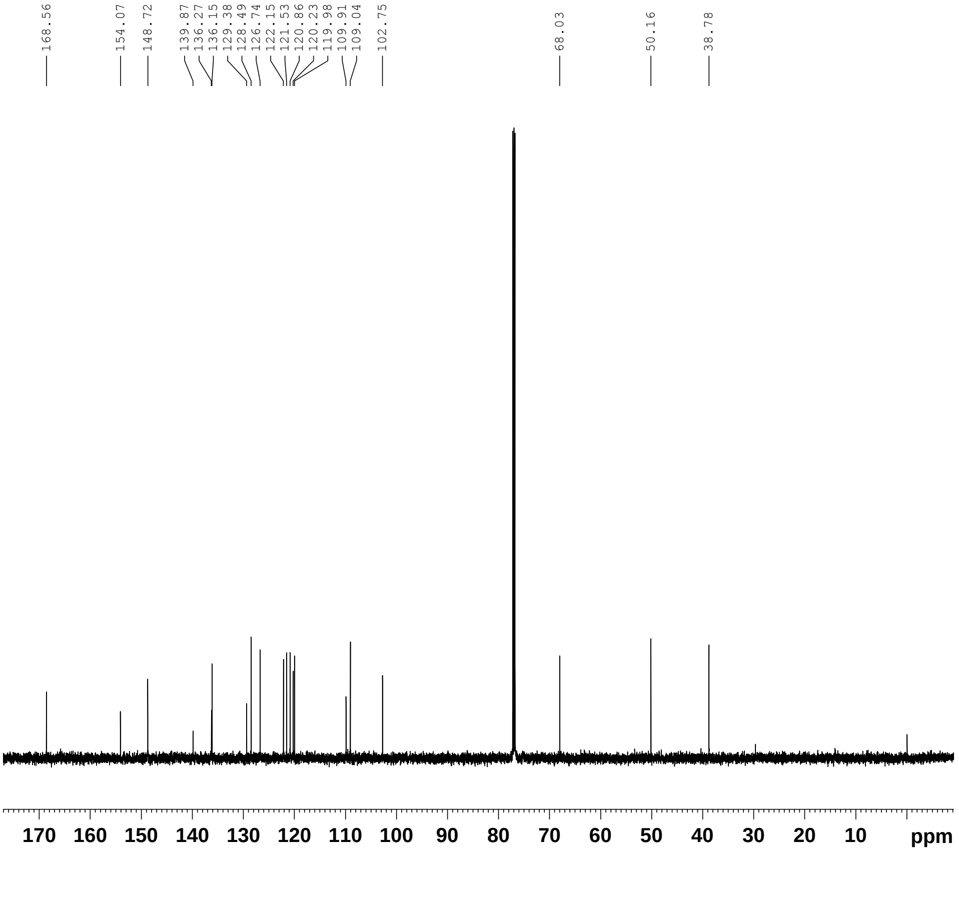

NAME FJ03902
EXPNO 12
PROCNO 1
Date_ 20170518
Time 20.52 h
INSTRUM spect
PROBHD Z847801_0047 (
PULPROG zgdc30
TD 32768
SOLVENT CDCl3
NS 128
DS 0
SWH 36057.691 Hz
FIDRES 2.200787 Hz
AQ 0.4544329 sec
RG 2050
DW 13.867 usec
DE 6.50 usec
TE 293.0 K
D1 1.50000000 sec
D11 0.03000000 sec
TD0 1
SFO1 150.8892338 MHz
NUC1 13C
P1 9.80 usec
SI 65536
SF 150.8726448 MHz
WDW EM
SSB 0
LB 1.00 Hz
GB 0
PC 1.40

Compound 8:

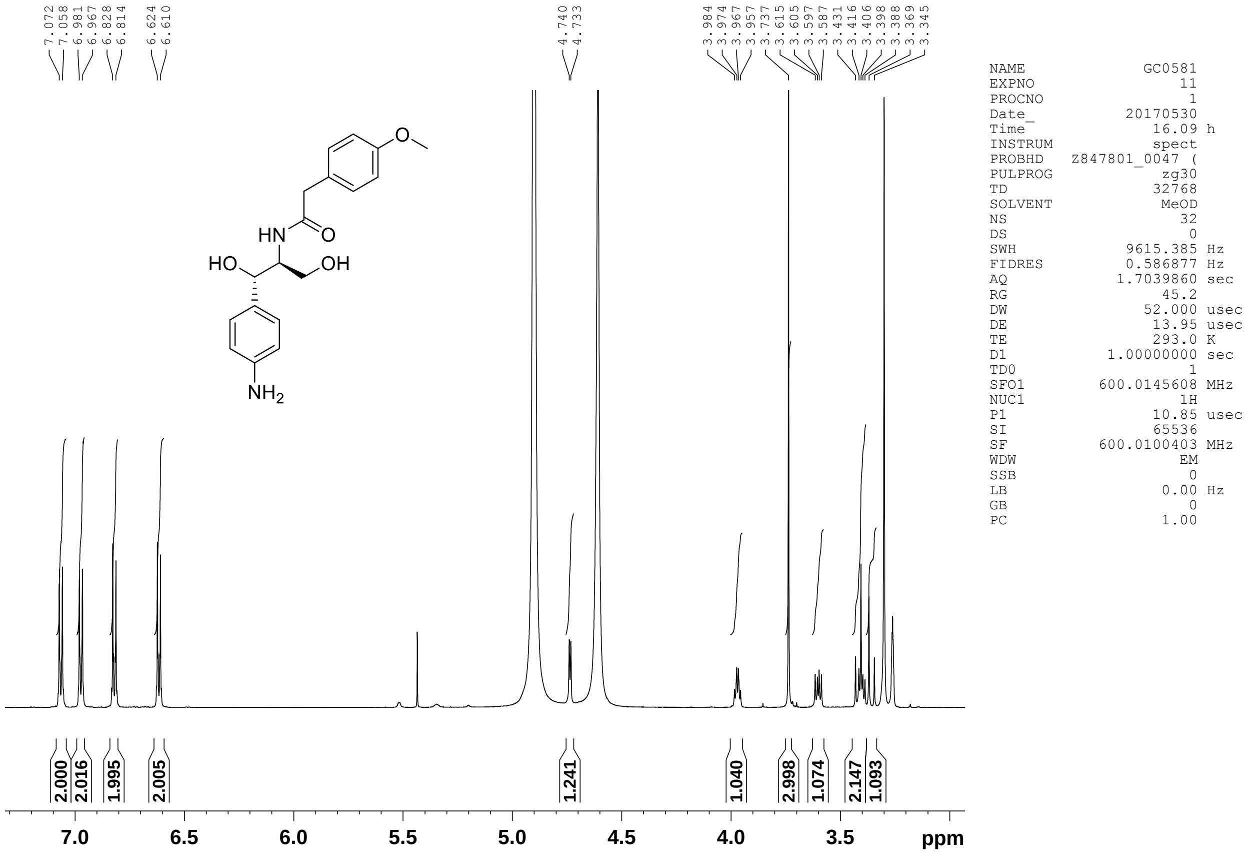
7.072
7.058
6.981
6.967
6.828
6.814
6.624
6.610
4.740
4.733
3.984
3.974
3.967
3.957
3.737
3.615
3.605
3.597
3.587
3.431
3.416
3.406
3.398
3.388
3.369
3.345
HN
O
HO
OH
NH2
O
2.000
2.016
1.995
2.005
1.241
1.040
2.998
1.074
2.147
1.093
7.0
6.5
6.0
5.5
5.0
4.5
4.0
3.5
ppm
NAME GC0581
EXPNO 11
PROCNO 1
Date_ 20170530
Time 16.09 h
INSTRUM spect
PROBHD Z847801_0047 (
PULPROG zg30
TD 32768
SOLVENT MeOD
NS 32
DS 0
SWH 9615.385 Hz
FIDRES 0.586877 Hz
AQ 1.7039860 sec
RG 45.2
DW 52.000 usec
DE 13.95 usec
TE 293.0 K
D1 1.00000000 sec
TD0 1
SFO1 600.0145608 MHz
NUC1 1H
P1 10.85 usec
SI 65536
SF 600.0100403 MHz
WDW EM
SSB 0
LB 0.00 Hz
GB 0
PC 1.00

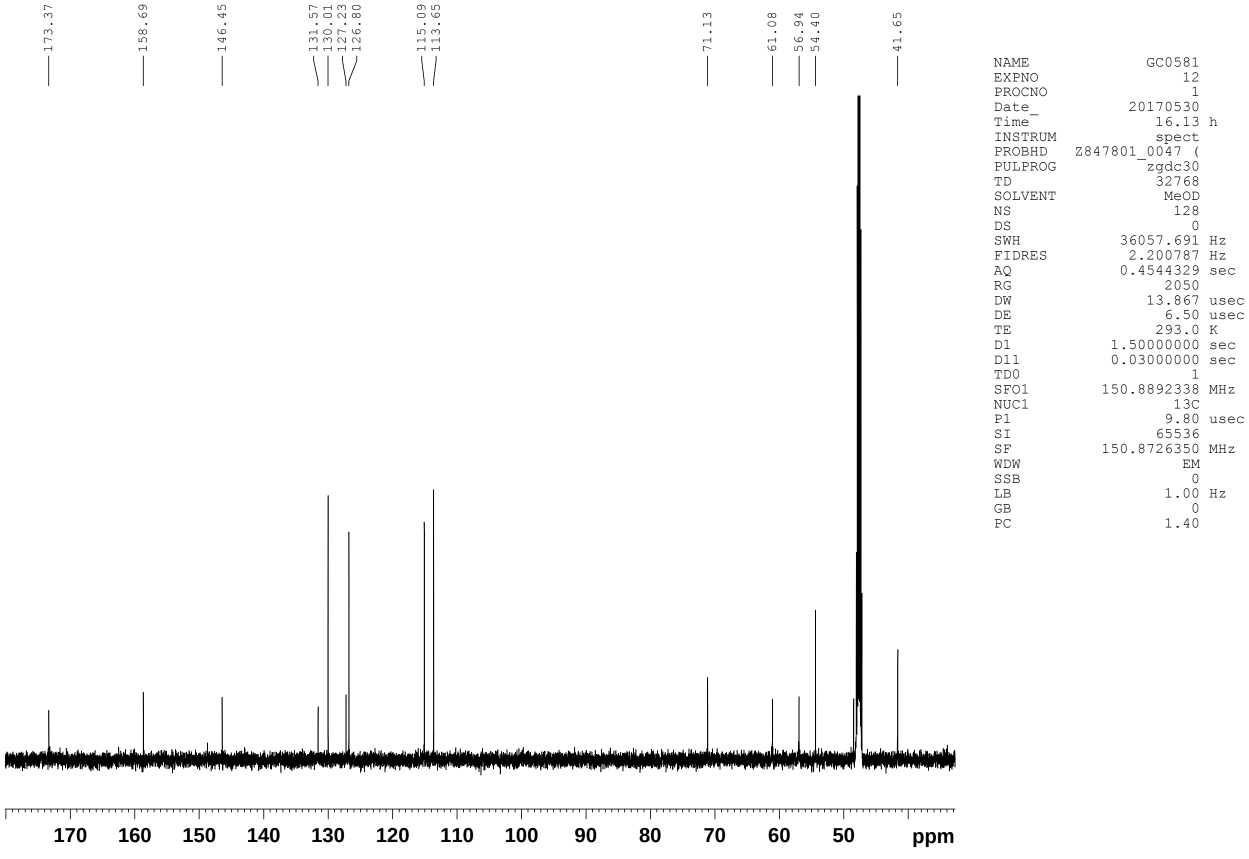
173.37
158.69
146.45
131.57
130.01
127.23
126.80
115.09
113.65
71.13
61.08
56.94
54.40
41.65
170
160
150
140
130
120
110
100
90
80
70
60
50
ppm
NAME GC0581
EXPNO 12
PROCNO 1
Date_ 20170530
Time 16.13 h
INSTRUM spect
PROBHD Z847801_0047 (
PULPROG zgdc30
TD 32768
SOLVENT MeOD
NS 128
DS 0
SWH 36057.691 Hz
FIDRES 2.200787 Hz
AQ 0.4544329 sec
RG 2050
DW 13.867 usec
DE 6.50 usec
TE 293.0 K
D1 1.50000000 sec
D11 0.03000000 sec
TD0 1
SFO1 150.8892338 MHz
NUC1 13C
P1 9.80 usec
SI 65536
SF 150.8726350 MHz
WDW EM
SSB 0
LB 1.00 Hz
GB 0
PC 1.40
